# Forcing Single‐Column Models Using High‐Resolution Model Simulations

**DOI:** 10.1029/2017MS001189

**Published:** 2018-08-06

**Authors:** Hannah M. Christensen, Andrew Dawson, Christopher E. Holloway

**Affiliations:** ^1^ Atmospheric, Oceanic and Planetary Physics University of Oxford Oxford UK; ^2^ National Center for Atmospheric Research Boulder CO USA; ^3^ Department of Meteorology University of Reading Reading UK

**Keywords:** single‐column model, forcing data, high‐resolution modeling, geostrophic winds, Indian Ocean/warm pool, NERC Cascade project

## Abstract

To use single‐column models (SCMs) as a research tool for parameterization development and process studies, the SCM must be supplied with realistic initial profiles, forcing fields, and boundary conditions. We propose a new technique for deriving these required profiles, motivated by the increase in number and scale of high‐resolution convection‐permitting simulations. We suggest that these high‐resolution simulations be coarse grained to the required resolution of an SCM, and thereby be used as a proxy for the true atmosphere. This paper describes the implementation of such a technique. We test the proposed methodology using high‐resolution data from the UK Met Office's Unified Model, with a resolution of 4 km, covering a large tropical domain. These data are coarse grained and used to drive the European Centre for Medium‐Range Weather Forecast's Integrated Forecasting System (IFS) SCM. The proposed method is evaluated by deriving IFS SCM forcing profiles from a consistent T639 IFS simulation. The SCM simulations track the global model, indicating a consistency between the estimated forcing fields and the true dynamical forcing in the global model. We demonstrate the benefits of selecting SCM forcing profiles from across a large domain, namely, robust statistics, and the ability to test the SCM over a range of boundary conditions. We also compare driving the SCM with the coarse‐grained data set to driving it using the European Centre for Medium‐Range Weather Forecast operational analysis. We conclude by highlighting the importance of understanding biases in the high‐resolution data set and suggest that our approach be used in combination with observationally derived forcing data sets.

## Key Points

- We propose and implement a methodology for deriving single‐column model (SCM) forcing data sets from large‐domain high‐resolution convection‐permitting simulations
- The large spatial domain samples many weather regimes and boundary conditions and allows for robust conclusions to be drawn
- We demonstrate improvements over deriving forcing from an operational analysis, for which temporal interpolation introduces spurious features

## 1 Introduction

Single‐column models (SCMs) are widely used to understand physical processes and to inform the development of parameterization schemes. An SCM takes a single atmospheric column from a parent general circulation model (GCM). This column contains the physical parameterization schemes that are used in the GCM to represent unresolved subgrid‐scale processes, while the model dynamics is replaced with boundary forcings. It is computationally cheap to run a single atmospheric column instead of the full GCM. An SCM therefore provides one important means of testing new parameterization schemes and validating these new schemes over a range of cases, as hundreds or thousands of simulations may be performed to test and tune the scheme. It is also possible to use SCMs under idealized forcing scenarios to develop fundamental understanding about the atmosphere, such as understanding radiative‐convective equilibrium (Emanuel et al., 2014). SCMs have long been used for these purposes (Betts & Miller, 1986; Randall et al., 1996), and are still widely used today in the international community: for example, the Global Energy and Water Cycle Experiment Cloud Systems Study recommends SCMs as a key tool for modeling and understanding cloud systems (Bechtold et al., 2000; Petch et al., 2007). A key limitation of using an SCM is the lack of two‐way interaction between the large‐scale circulation and the parameterized physics processes. This lack of two‐way interaction can lead to spurious behavior, such as oscillating between states or locking into a state, which does not happen when using a full GCM. Nevertheless, SCMs have an important place in model development as one evaluation technique among a hierarchy of approaches (Randall et al., 1996).

A key challenge in using SCMs is deriving the required forcing data, which includes the initial conditions and advected tendencies of the prognostic variables. The canonical technique is to use forcing data sets derived from observations (Randall & Cripe, 1999), such as data from the Atmospheric Radiation Measurement program (Ackerman & Stokes, 2003; Turner & Ellingson, 2016). The evolution of the SCM can then be directly compared to the observed data set. Furthermore, the same observed forcing can be used to drive high‐resolution cloud resolving models (CRMs), and the representation of subgrid processes in the SCM and CRM can be compared (Randall et al., 1996).

To derive SCM forcing data sets in this way, high‐quality observational data is required. An array of observation platforms is required to estimate the advective fluxes of prognostic variables, including balloon soundings and surface instruments (Zhang et al., 2016). There are only a handful of sites globally with this capability. The forcing data should have a high temporal frequency. Only during intensive operational periods are balloon measurements made at 3‐hourly (or 6‐hourly) frequency, though surface‐based measurements of wind profiles, turbulent surface fluxes, and precipitation are available with a higher frequency (Zhang et al., 2016).

In order to derive the observational forcing data sets, assumptions and approximations must be made, which can affect the final analysis (Zhang et al., 2001). For example, two separate groups derived analyses for the Tropical Ocean and Global Atmosphere Coupled Ocean‐Atmospheric Response Experiment. The different derivation process led to differences in the moisture budget: the average precipitation over the period was reported as 5.7–6.1 mm/day by X. Lin and Johnson (1996) and as 10.5–11.8 mm/day by Frank et al. (1996). New sophisticated methods have been developed to reduce errors in the large‐scale forcing (Zhang & Lin, 1997; Zhang et al., 2001), though the accuracy of the data can be limited by missing data and scale aliasing in the measurements. In addition, recent advances have seen the development of a three‐dimensional constrained variational analysis approach (Tang & Zhang, 2015), which enables the estimation of fine‐resolution SCM forcing data sets from observational data. The new technique enables the estimation of atmospheric profiles for a number of adjacent columns around suitable observational sites. These columns can then be aggregated to the desired resolution to evaluate scale‐aware parameterizations or used to force several adjacent SCMs to consider the impact of small‐scale variability (Tang et al., 2017).

It is also possible to use reanalysis products to derive the required forcing fields. Such products have the required temporal and spatial frequency and can be produced for long periods at any global location. However, the models used to produce the reanalysis products can exhibit large biases in the representation of clouds and precipitation: the operational European Centre for Medium‐Range Weather Forecasting (ECMWF) analysis has resolution of order 50 km, so these processes are parameterized. Xie et al. (2003) demonstrate large differences between SCM forced by observations and those forced by (re)analysis products, which they relate to errors in the ECMWF model's representation of clouds and precipitation. However, Xie et al. (2004) demonstrate that surface and top of atmosphere budget constraints can be used to substantially improve the forcing derived from operational analysis data, rendering it suitable for budget analysis and cloud studies.

Recent years have seen an increase in the production of large‐domain, convection‐resolving (or at least convection‐permitting) atmospheric simulations, including both CRMs with resolutions of a few kilometers (Holloway et al., 2012) and large eddy simulations (LES) with resolutions of order 100 m. These simulations cover large domains. For example, the *Cascade* project produced high‐resolution convection‐permitting simulations using the UK Met Office Unified Model (MetUM) at 1.5‐km and 4‐km resolution over a domain covering most of West Africa, and a domain spanning 15,500 km by 4,500 km over the Indian Ocean, Warm Pool and tropical west Pacific (Holloway et al., 2013; Marsham et al., 2011), while the High‐Definition Clouds and Precipitation for advancing Climate Prediction [HD(CP)^2^] project used the ICOsahedral Nonhydrostatic atmosphere model for a LES over Germany at 156‐m resolution for 4 days (Heinze et al., 2017). The increase in production of extensive high‐resolution data sets (Heinze et al., 2017; Holloway et al., 2012; Satoh et al., 2014, 2017; Schalkwijk et al., 2015) has largely become possible due to computational advances in recent years.

The availability of these high‐resolution data sets suggests a new possibility for furthering parameterization development within the SCM framework. Unlike the traditional partnership between LES and SCM, whereby observations are used to drive both the SCM and a LES that covers the same region (Randall et al., 1996), we propose the use of large‐domain high‐resolution simulations to derive the forcing data sets required by the SCM. This allows for *perfect model* studies to be carried out, whereby the high‐resolution data take the place of observations, and become the target that the parameterized SCM seeks to match.

This approach has several advantages over using observations. First, the high‐resolution data sets cover a large spatial area. This allows for parameterization schemes to be tested across many climate and weather regimes and over a wide range of land/sea boundary conditions. It is also increasingly the case that high‐resolution data sets have excellent temporal coverage, allowing such simulations to rival the observational data from intensive operational periods. In addition, CRMs are able to simulate certain aspects of the climate system better than GCMs, including the distribution of precipitation and the diurnal cycle of convection, providing benefits over analysis‐derived data sets.

Evaluating SCMs in a perfect model framework can simplify the evaluation process, as all model fields are available for comparison (Bechtold et al., 2000). This includes quantities that are hard to measure, such as the vertical distribution of liquid water and ice and the in‐cloud temperature (Noh et al., 2013). However, the perfect model framework also has drawbacks. For example, convection‐resolving or convection‐permitting models will be biased with respect to observations, including biases in the representation of convective processes. Care must be taken to distinguish biases in the SCM from biases in the CRM. Fortunately, biases in existing CRM simulations have been extensively documented (e.g., Holloway et al., 2012, 2013), facilitating this procedure.

In this paper, we discuss the feasibility of using high‐resolution atmospheric simulations as an alternative to observations for driving an SCM. The aims of this paper are threefold. First, we describe the process of coarse graining an existing high‐resolution simulation to derive initial condition and forcing fields for a single‐column model. We then evaluate the validity of forcing an SCM using such coarse‐grained data, before we demonstrate some of the benefits of using data from CRM for SCM studies. To illustrate the procedure, we use data from the Cascade project produced using the MetUM at 4‐km resolution. To demonstrate the generality of the procedure, we use these data to derive forcing data sets for the ECMWF Integrated Forecasting System (IFS) SCM. This paper therefore focuses on the implementation of such a methodology, while applications will be discussed in future manuscripts.

The paper is organized as follows. In section 2 we describe the Cascade data set, while in section 3 and Appendix A we describe the IFS SCM. In section 4 we describe the coarse‐graining procedure, whereby the high‐resolution data are used to derive initial condition and forcing data at the chosen resolution of the SCM. In section 5 we perform several tests to validate the derived forcing files, including evaluating the impact of coarse‐graining assumptions on the diagnosed biases and validation of the estimated geostrophic wind forcing. In section 6 we demonstrate advantages of the proposed technique, including consideration of regime‐dependent model biases and comparison of the behavior of SCM when using the derived forcing files with that using ECMWF operational analysis. Finally, we make some concluding remarks in section 7.

## 2 The High‐Resolution Model Simulation

The high‐resolution model simulation used here was produced as part of Cascade, a UK consortium project funded by the Natural Environment Research Council (NERC). The Cascade project studied the interaction between tropical convection and large‐scale processes including the diurnal cycle, the Madden‐Julian oscillation (MJO), and equatorially trapped waves (Holloway et al., 2013; Love et al., 2011; Pearson et al., 2010). To do so, it produced convection‐permitting, cloud system‐resolving simulations of the UK Met Office Unified Model (UM) with resolutions from 1.5 km to 12 km over several large tropical domains. These simulations were compared to simulations of the same model with parameterized convection and to observational data sets.

For this paper, we use the Cascade 4‐km resolution tropical Indo‐Pacific Warm Pool integration. For full details of the simulation, see Holloway et al. (2012). In brief, the simulations were produced using the limited‐area setup of the MetUM version 7.1 (Davies et al., 2005). The model is semi‐Lagrangian and nonhydrostatic, and the 4‐km simulation covers the domain 20^∘^S–20^∘^N, 42^∘^E–177^∘^E. Initial conditions were specified from the ECMWF operational analysis. This analysis was produced using four‐dimensional variational data assimilation, with outer loop resolution of T799 (25 km). The inner loop resolution was initially T95 (210 km) for efficiency, before increasing to T255 (80 km). A 12‐km parameterized convection run was first produced over a domain 1^∘^ larger in each direction, with lateral boundary conditions relaxed to the ECMWF operational analysis. The 4‐km run was forced using lateral boundary conditions computed from the 12‐km parameterized run, via a nudged rim of eight model grid points. The model has 70 terrain‐following hybrid levels in the vertical, with vertical resolution ranging from tens of meters in the boundary layer to 250 m in the free troposphere, and with model top at 40 km. The time step was 30 s.

At 4‐km resolution, the model is *convection permitting*: the convectively available potential energy (CAPE)‐closed convection scheme of Gregory and Rowntree (1990) is adapted to have a very long CAPE timescale at large CAPE values, such that at large CAPE values the convection scheme is effectively *turned off*, and almost all rainfall is generated explicitly. The chosen simulation uses Smagorinsky subgrid mixing in the horizontal and vertical dimensions. The simulation begins on 6 April 2009 and spans 10 days, chosen as a case study of an active MJO event. Data from the Cascade project is available on request from the NERC Centre for Environmental Data Analysis. The data are stored at full resolution in space and once an hour in time.

Thorough validation of the Cascade simulation has been reported by Holloway et al. (2012, 2013, 2015). The 4‐km Cascade simulation with 3‐D Smagorinsky mixing is substantially more realistic than simulations with parameterized convection. The simulation shows a realistic vertical heating structure (Holloway et al., 2015) and has a precipitation distribution that is similar to that diagnosed from Tropical Rainfall Measuring Mission observations (Holloway et al., 2012). This is likely due to improved profiles of moist static energy and saturation moist static energy compared to parameterized models (Holloway et al., 2012). The Cascade simulation also displays a good relationship between precipitation rate and tropospheric humidity (Holloway et al., 2012, 2013), and realistic generation of eddy available potential energy (Holloway et al., 2013). This allows for a realistic MJO propagation, such that the degree of convective organization, MJO strength, and propagation speed match those observed (Holloway et al., 2013). The model has a realistic representation of vertical and zonal wind speeds compared to ECMWF operational analysis, though the large‐scale ascent is less confined than in observations (Holloway et al., 2013). However, the simulation does not conserve moisture, such that the precipitation rate is 8% higher than predicted by the other terms in the moisture budget (Holloway et al., 2015). This can be traced to the advection scheme creating spurious rainfall, as shown in Holloway et al. (2015). Fortunately, this problem does not contribute significantly to heating, so a sufficient solution is to scale the Cascade rainfall down when comparing with SCM simulations. The first day of the Cascade simulation showed a very strong spin‐up, especially in the precipitation flux (Holloway et al., 2012). For this reason, we discard the first day of simulation, and use the remaining 9 days to derive forcing fields for the IFS SCM.

## 3 The IFS Single‐Column Model

The IFS SCM is based on the code for the full IFS model and includes the atmospheric physics parameterizations and land surface scheme. The SCM has been released through the OpenIFS project and so is freely available to researchers and educational institutions on application for an OpenIFS license. The IFS SCM used here is based on IFS Cycle 40R1. The full reference and user's guide (Köhler & Teixeira, 2002) can be obtained on request from *openifs‐support@ecmwf.int*. Certain details are reproduced here for convenience.

The IFS SCM requires a set of initial conditions for the atmosphere, together with a set of boundary conditions describing aspects of the lower boundary including orography, vegetation types and covers, and sea surface temperature. The land surface scheme can be replaced by specifying surface‐sensible and latent heat fluxes with a consistent skin temperature. Table A1 in Appendix A contains an exhaustive list of boundary conditions and prognostic variables for which initial conditions are required.

The IFS SCM contains only simplified dynamic equations. A number of external forcing fields are specified, including the vertical velocity, geostrophic winds, and advective tendencies of zonal and meridional winds, temperature, and specific humidity (see Table A1). In addition, the prognostic variables can be relaxed toward specified observed fields. The equations of motion solved by the IFS are given in Appendix A and compared to those solved by the IFS SCM. This allows the dynamical source of each of the SCM forcing terms to be identified. In particular, the horizontal pressure term in the momentum equations is supplied in terms of a geostrophic wind forcing.

## 4 Coarse Graining the Cascade Data Set

We interpret the IFS SCM as representing the evolution of a single column of the ECMWF global IFS model. The forcing fields applied to the SCM should represent the forcings that would be experienced by the parameterization schemes when used in the full global model, that is, at a resolution representative of the global model. In this study, the resolution of the SCM was chosen to be that of the operational IFS ensemble forecasting system model at the time of writing, namely, 91 vertical levels with a horizontal resolution of T639, which corresponds to approximately 0.28^∘^ or 30‐km grid spacing. The reduced Gaussian grid used for the physical parameterization schemes in the operational IFS was used to define the latitude and longitude coordinates within the Cascade domain of 20^∘^S to 20^∘^N, 42^∘^E to 177^∘^E. An Eulerian time‐stepping scheme is used, with a time step of 15 min.

### 4.1 Producing the Initial Condition Fields

In general, the fields from the Cascade simulation are first linearly interpolated to give values every 15 min, before being coarsened to the SCM grid. We note that the temporal interpolation is orthogonal to spatial averaging, such that the coarse‐grained results are insensitive to the order in which these two operations are performed. Spatial coarse graining is achieved using local area averaging (Porta Mana & Zanna, 2014). This accounts for the contribution of fractional high‐resolution grid boxes to the low‐resolution average:

(1) $${\bar{\psi }}_{n,k}=\overset{I}{\underset{i}{\sum }}W_{n,i}\psi_{i,k}$$

where *ψ*_*i*_ denotes the field on the fine grid and ${\bar{\psi }}_{n}$ denotes the field on the coarsened grid. The index *n* identifies the coarse grid box, while the index *i* identifies the fine grid. *W*_*n*,*i*_ is an areal weighting function indicating the fraction of fine grid box *i* within coarse grid box *n*. The index *k* denotes the vertical level of the field.

#### 4.1.1 Sensitivity to Vertical Interpolation Procedure

Both the fine‐ and coarse‐resolution data sets are defined on model levels. To produce the coarse‐grained data set, we first evaluate the coarse‐scale grid box mean surface pressure, $\bar{p_{s}}$, by averaging the fine‐scale surface pressure within each coarse grid box following equation (1). The low‐resolution surface pressure field is then combined with the ECMWF hybrid height coefficients, *A*_*k*_ and *B*_*k*_, to define the pressure on the ECMWF hybrid model levels (both full and half):

(2) $$p=A(\eta )+B(\eta )p_{s}$$

where the hybrid *A* and *B* coefficients have been written in terms of the model level coordinate *η*. Having defined the required output pressure levels as a function of horizontal position, the question remains of how to perform horizontal averaging and vertical interpolation, particularly in regions of topography, where the input pressure levels on the fine grid change from grid box to grid box. This is a philosophical question, concerning how to interpret the variables on a coarse‐grained grid in regions of topography. We consider two possible options:

1. Since the coarse‐grained surface pressure is the average of the fine‐scale surface pressure, the coarse‐grained surface temperature should be the average of the fine‐scale surface temperature and so on. Therefore, first, average all input variables horizontally along each model level to produce the low‐resolution variable on that level. Interpolate these area‐averaged fields, logarithmically in pressure, from their native model levels to the required ECMWF *η* levels.
2. The coarse‐grained variables represent the average of the fine‐scale fields on the pressure levels defined by the coarse grid. Therefore, first, interpolate the fine‐scale fields, logarithmically in pressure, from their native model levels to the required ECMWF *η* levels for each coarse‐grained grid box. Average these fields horizontally along pressure levels.

It is not clear which method is preferable, as each method has its drawbacks. Method (1) involves averaging variables across different pressures (heights above sea level), following the local topography. On the other hand, Method (2) involves averaging variables across different heights above the surface and requires extrapolation of fields to levels below the ground for variables upslope of the center of the coarse grid box. A further (algorithmic) drawback of Method (2) is that, for a general pair of grids, there is not a one‐to‐one mapping between the fine grid boxes and the coarse grid boxes: a fine grid box may contribute to more than one coarse grid box following equation (1). Such fine grid columns must therefore be interpolated to the coarse‐grained pressure levels associated with more than one coarse grid box, before being horizontally averaged. Both methods will be first tested over a limited domain to assess sensitivities to the coarse‐graining procedure and to choose the preferred technique.

Furthermore for both methods,

1. before interpolation and averaging, the upward air velocity in the Cascade data set is converted from *w*/(ms^−1^) to *ω*/(Pas^−1^) using the relationship $\omega =-\frac{\mathrm{wpg}}{\mathrm{RT}}$, where *g* is the acceleration due to gravity and the other symbols are as defined earlier. Note that the Cascade data are produced using a nonhydrostatic model, run at fairly high resolution, so using the hydrostatic approximation may introduce some small errors.
2. The water vapor, liquid water, and ice water variables in Cascade must be converted from specific humidities, *q* = *ρ*_*v*_/(*ρ*_*v*_+*ρ*_*d*_) to the mixing ratios required by the IFS SCM, *q* = *ρ*_*v*_/*ρ*_*d*_, where the total density of air, *ρ* is given as a sum of the density of dry and moist air (*ρ*_*d*_ and *ρ*_*v*_ respectively). The area average of each specific humidity is first evaluated before being converted to a mixing ratio. This ensures conservation of moist static energy (n.b., the difference between $\bar{\left(\frac{\rho_{v}}{\rho_{d}}\right)}$ and $\frac{\bar{\rho_{v}}}{\bar{\rho_{d}}}$ is of the order of 1,000th of a percent in the mean, with a maximum local value of 10^−4^%).
3. The cloud fraction in Cascade is stored as four separate fields: area fractions of large‐scale ice cloud, large‐scale water cloud, convective‐scale ice cloud, and convective‐scale water cloud, respectively. The sum of these fields, *s*, follows 0≤*s*≤1.The four fields are added before areal averaging and vertical interpolation take place.

The Cascade model top is at 40‐km altitude. Above this level, the fields are padded using ECMWF operational analysis data. The Cascade and analysis data are blended over five model levels to ensure a smooth transition. A weighted average is performed such that the final field, *F* = *x*.*F*_cas_+*y*.*F*_an_, where *F*_cas_ and *F*_an_ are the Cascade and analysis fields, respectively, and (x,y) = (0.2,0.8), (0.4,0.6), (0.6,0.4), (0.8,0.2), (1.0,0.0) for the five blended levels, respectively. The physical parameterizations of interest are localized at altitudes well below 40 km, so this blending should have only a small impact on the SCM simulation.

The high‐resolution Cascade data set exhibits pervasive resolved gravity waves across the domain at all times, as well as other small‐scale features. On coarse graining to the IFS resolution, the gravity waves are no longer resolved and appear as grid‐scale noise. Such high‐frequency waves would not be a solution of the lower‐resolution equations at T639, and instead, their impact would be parameterized. We therefore apply a nine‐point Gaussian smoother to all initial condition fields after coarse graining to remove fine‐scale features such as these. All derived forcing fields (described below) are evaluated using these smoothed fields.

### 4.2 Producing the Boundary Fields

The boundary fields listed in Table A1 were extracted from the ECMWF archive at the desired resolution for the SCM (i.e., T639). This ensures that the SCM will have the same boundary conditions as the operational IFS model.

The archived Cascade simulation data does not include soil moisture or temperature variables. Therefore, the interactive land surface processes are turned off in the IFS SCM, and time‐varying, surface‐sensible and latent fluxes, and skin temperature are provided from the Cascade simulation instead. These three fields were linearly interpolated in time before they were areally averaged following equation (1), as for the initial condition fields. Over land points the sea surface temperature field is set to the skin temperature, as the model requires initialization of all fields.

### 4.3 Producing the Forcing Fields

In the IFS SCM, all forcing fields are assumed instantaneous. Forcing fields must be derived for the horizontal advective tendencies, the vertical velocity in model coordinates, $\dot{\eta }\frac{\mathrm{\partial p}}{\partial \eta }$, and the geostrophic winds.

#### 4.3.1 Advective Tendencies

The IFS SCM must be forced using the advected tendencies of temperature (*T*), humidity (*q*), and horizontal wind components (*u* and *v*). These tendencies are quasi‐horizontal, and so evaluated along terrain‐following model levels. Note that in this framework horizontal advection of other variables, including hydrometeors, can also be calculated, if the chosen SCM is able to take such tendencies as input (which the IFS SCM is not). In general, hydrometeor tendencies are missing from current SCM data sets due to lack of suitable observations.

To derive the SCM advective tendencies, we must understand the relationship between the coarse‐scale and fine‐scale forcing fields. On the fine grid, assuming incompressibility, the variables evolve following

(3) $$\frac{\partial \psi_{i,k}}{\mathrm{\partial t}}+u_{i,k}\cdot \nabla_{k}(\psi_{i,k})=F_{i,k}$$

where ∇_*k*_ is the gradient operator on the fine grid along model level k, and *F*_*i*,*k*_ is the subgrid‐scale forcing on the fine grid. Consider coarse graining this equation following equation (1):

(4) $$\frac{\partial {\bar{\psi }}_{n,k}}{\mathrm{\partial t}}+{\bar{\left(u_{i,k}\cdot \nabla_{k}(\psi_{i,k})\right)}}_{n}={\bar{F}}_{n,k}$$

Rearranging,

(5) $$\frac{\partial {\bar{\psi }}_{n,k}}{\mathrm{\partial t}}+\underset{A}{\underbrace{{\bar{u}}_{n,k}\cdot {\bar{\nabla }}_{k}({\bar{\psi }}_{n,k})}}=\underset{B}{\underbrace{{\bar{u}}_{n,k}\cdot {\bar{\nabla }}_{k}({\bar{\psi }}_{n,k})-{\bar{\left(u_{i,k}\cdot \nabla_{k}(\psi_{i,k})\right)}}_{n}+{\bar{F}}_{n,k}}}$$

Term A on the left‐hand side of the equation is the advective tendency as represented on the coarse‐grained grid, where ${\bar{\nabla }}_{k}$ is the gradient operator on the coarse grid along model level *k*. This term is required to force the IFS SCM. The terms B on the right‐hand side contain contributions from two sources. The first two terms in B represent the contribution of processes that were resolved by the finer grid but are now unresolved on the coarse grid. The final term represents the contribution from processes that were unresolved on the finer grid and remain unresolved on the coarse grid: only the average of these small‐scale processes, ${\bar{F}}_{n,k}$, must be represented to capture the evolution of the coarse‐grained prognostic variables. Taken together, the terms B are equal to the subgrid‐scale forcing on the coarse grid (Porta Mana & Zanna, 2014; Shutts & Palmer, 2007). While each of these processes will not be represented separately, the combined impact of terms B should be represented by the IFS parameterization schemes.

We, therefore, derive the advective tendencies not from the Cascade tendencies archived on the fine‐scale grid as might be naively expected, but instead from the coarsened fields of $\bar{u}$, $\bar{v}$, $\bar{T}$, and $\bar{q}$. The advective tendency is given:

(6) $$\text{adv}(\psi ){|}_{n,k}=-{\bar{u}}_{n,k}\cdot {\bar{\nabla }}_{k}(\bar{\psi_{n,k}})$$

for variable *ψ*. The vector gradient in *ψ* is first estimated using a centered finite difference scheme before the dot product is taken with the coarse‐grained vector wind field.

#### 4.3.2 Vertical Velocity

The pressure in model level coordinates as given by equation (2) allows us to expand the vertical pressure velocity, $\dot{\eta }\frac{\mathrm{\partial p}}{\partial \eta }$, in terms of model vertical coordinates:

(7) $$\omega =\frac{dp}{dt}=u_{k}\cdot \nabla_{k}p+{\left.\dot{\eta }\frac{\mathrm{\partial p}}{\partial \eta }\right|}_{p_{s}}{\left.+\frac{\mathrm{\partial p}}{\partial p_{s}}\right|}_{\eta }\frac{dp_{s}}{dt}{\left.+\frac{\mathrm{\partial p}}{\mathrm{\partial t}}\right|}_{\eta ,p_{s}}$$

Therefore,

(8) $${\left.\dot{\eta }\frac{\mathrm{\partial p}}{\partial \eta }\right|}_{p_{s}}=\omega -u_{k}\cdot \nabla_{k}p-B(\eta )\frac{dp_{s}}{dt}$$

using the definition of *B* in equation (2) and recognizing that ${\left.\frac{\mathrm{\partial p}}{\mathrm{\partial t}}\right|}_{\eta ,p_{s}}=0$. Equation (8) is evaluated using the coarse‐grained fields on the full model levels, before it is interpolated logarithmically in pressure coordinates to the half levels. The first and last model half level represent the higher and lower bounds of the model respectively: the vertical velocity on these levels is set to zero.

#### 4.3.3 Geostrophic Winds

The geostrophic winds used to force the IFS SCM are given as

(9) $$fv_{g}={\left.\frac{\partial \phi }{\mathrm{\partial x}}\right|}_{\eta }+{\left.\frac{1}{\rho }\frac{\mathrm{\partial p}}{\mathrm{\partial x}}\right|}_{\eta }$$

(10) $$fu_{g}=-\left.{\left(\frac{\partial \phi }{\mathrm{\partial y}}\right|}_{\eta }+\frac{1}{\rho }{\left.\frac{\mathrm{\partial p}}{\mathrm{\partial y}}\right|}_{\eta }\right),$$

where the first term in each equation is due to the gravitational forcing along the terrain‐following model levels, and the second term is the pressure gradient term along model levels. In regions with orography the two terms in each geostrophic equations (9) and 10 are large and almost exactly compensate each other due to the vertical pressure gradient. The small residual gives the geostrophic wind. Numerical limitations can therefore make it difficult to estimate the geostrophic winds accurately in regions with orography (e.g., Danard et al., 1993; Haney, 1991). The larger the slope of the orography, the larger the potential error.

We note that the geostrophic winds are required by the IFS SCM to calculate the pressure forcing in the momentum equations (A7) and (A8). In tropical regions, such as those covered by Cascade, the geostrophic approximation is a poor representation of the observed wind field. However, we can still use the geostrophic equations to represent the pressure forcing, as required by the IFS SCM.

While we argued in section 4.2 that all boundary fields should be as used in the IFS, a key exception is the model orography/surface geopotential. Taking the surface geopotential from the Cascade simulation ensures consistency with the surface pressure field. Using consistent surface pressure and geopotential is especially important in the case of the IFS SCM, as there are noticeable features in the IFS surface geopotential field due to the spectral representation of the IFS. These spectral features adversely affect the geostrophic wind calculation, particularly as it is necessary to evaluate horizontal gradients.

To calculate the forcing for the IFS, we calculate the geostrophic winds following equations (9) and (10). The geopotential height is evaluated using the coarsened surface pressure, surface geopotential, and virtual temperature data from Cascade. The horizontal derivatives are evaluated using a centered finite difference scheme. The interpolated pressure force is divided by the Coriolis parameter to return the geostrophic winds.

**Figure 1:**
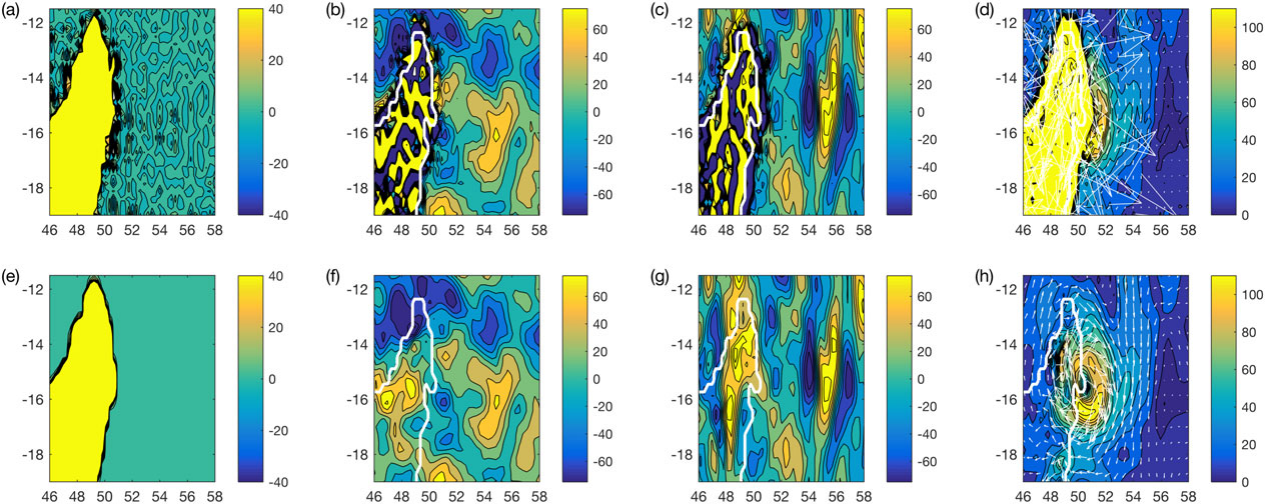
Example of geostrophic wind forcing diagnosed from Cascade data for a tropical cyclone over Madagascar, comparing the use of surface geopotential boundary fields from (top row) the IFS or (bottom row) the UM. Surface geopotential (m^2^/s^2^) in (a) the IFS and (e) the UM. The corresponding geostrophic winds in the zonal direction are shown in (b) and (f) for the IFS and UM, respectively, while the meridional components are shown in (c) and (g). The magnitude (color) and direction (arrows) of the geostrophic wind for the IFS and the UM are shown in (d) and (h), respectively. All wind speeds in meters per second. Note the spectral features in the IFS surface geopotential (panel a) can adversely affect the geopotential wind calculation. IFS = Integrated Forecasting System; UM = Unified Model.

To demonstrate the importance of using the Cascade surface geopotential for the geostrophic wind calculation, Figure 1 shows the surface geopotential field for both IFS and MetUM together with the geostrophic winds evaluated using these data, respectively. The spectral features in the IFS surface geopotential are clearly visible, together with the imprint on the geostrophic wind field.

## 5 Validation of the Derived Forcing

The total number of coarse‐grained grid points derived from the Cascade data set over which an SCM can be run is 480 in the longitudinal direction, and 142 latitudinally. With over 68,000 grid points available for evaluation of SCM simulations, robust statistics of bias can be estimated over a wide range of boundary and climatic conditions. In subsequent analysis, we consider three subregions: an area over the west Pacific (WP), an area over the Maritime Continent (MC), and a region west of Australia (WA). Each of these subregions has in excess of 2,800 grid points. The regions are summarized in Table 1.

**Table 1:** The Three Regions Selected From Within the Cascade Domain for Further Analysis

Region	Domain	Acronym	Comments
West Pacific	155^∘^E–178^∘^E, 5^∘^S–5^∘^N	WP	Only ocean points
Maritime Continent	100^∘^E–123^∘^E, 8^∘^S–2^∘^N	MC	Approximately 50% land, 50% ocean points
West of Australia	75^∘^E–105^∘^E, 18^∘^S–14.5^∘^S	WA	Only ocean points, little convective activity

Throughout the manuscript, we refer to the difference between the IFS SCM and the data set used to force it as a *bias*, highlighting that this is a systematic difference calculated by averaging over many grid points. However, since the methodology compares two models, this bias must be scrutinized before it can be attributed to model error in the SCM parameterization schemes: it is possible that the high‐resolution forcing data set has a bias compared to observations, which contributes to the difference between the SCM and the forcing data set.

### 5.1 Validation of Basic Philosophy

We first validate the foundational philosophy behind our approach, namely, that the evolution of the IFS SCM can be interpreted as equivalent to a single column taken from the global IFS model. In particular, we would like to evaluate whether estimating the SCM dynamical forcing from the instantaneous prognostic variables gives a good approximation to the true dynamical forcing in the global model. To assess this, we produce a global IFS forecast at T639 using the same model version as the SCM, CY40R1, where the atmospheric prognostic variables are archived on every model level once an hour. While the choice to archive the variables once an hour instead of every 15‐min time step was primarily based on available data storage, this is also consistent with the frequency of Cascade data. The three‐dimensional prognostic variables are used to calculate the required SCM dynamical forcing terms, namely, the advective tendencies, vertical pressure velocity, and geostrophic wind forcing. We also archive the temperature and moisture on each soil level, the skin temperature and the skin moisture, and the surface‐sensible and latent heat fluxes.

We expect the bias between the IFS SCM and the T639 global IFS simulation to be small. This does not indicate that the true biases in the SCM are small, but rather that the SCM shares biases with the global T639 IFS simulation, such that they evolve similarly in time. We compare two sets of SCM simulations over the MC domain forced using data from the T639 IFS simulation. In the first set of SCM simulations, the interactive land surface scheme is activated, and soil variables are also supplied from the global simulation instead of sensible and latent heat fluxes. In contrast, the second set of simulations uses supplied sensible and latent heat fluxes and skin temperature, and disables the land surface scheme. The global IFS forecast is consistent with the SCM, such that the SCM simulation will only diverge from the forcing data if the proposed methodology is unable to adequately supply the dynamical forcing, or if supplying fields at a 1‐hourly interval is insufficient to estimate the forcing on a 15‐min timescale.

**Figure 2:**
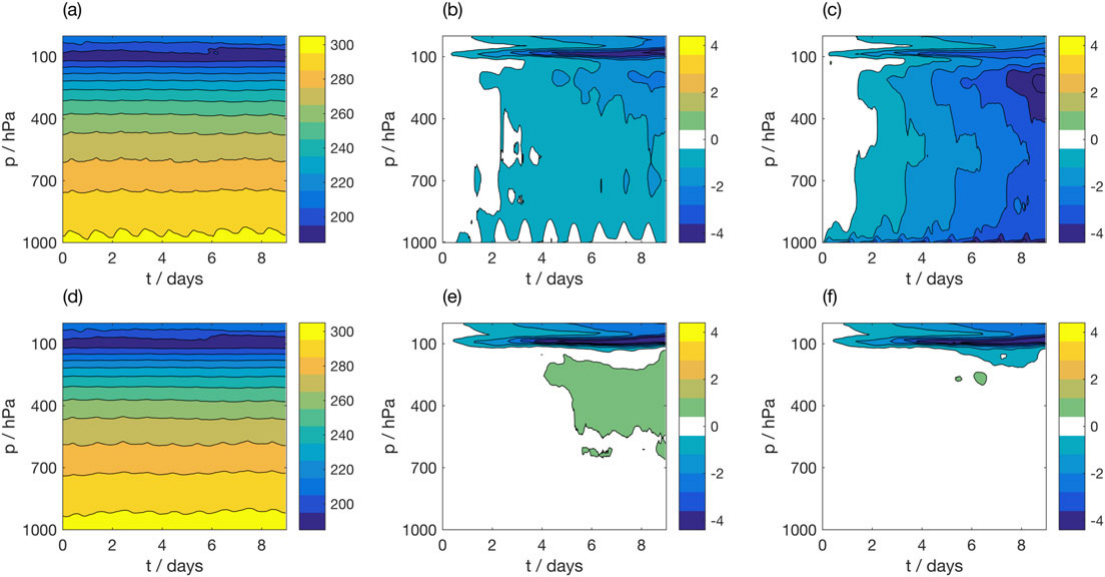
Evolution of temperature (K) as a function of height in global IFS simulations averaged over (a) land points and (d) ocean points in the Maritime Continent (MC) region. Bias in IFS SCM simulation over MC for the simulation forced using IFS data with (b, e) an interactive land scheme, over land and ocean points, respectively. and (c,f) the interactive land scheme replaced by forcing from sensible and latent heat fluxes, over land and ocean points, respectively. IFS = Integrated Forecasting System; SCM = single‐column model.

Figure 2 shows the evolution of SCM simulations in the MC region, forced using data from the consistent T639 IFS simulation. The top row shows the evolution of temperature over land points, while the bottom row shows the evolution over ocean points. The differences are higher over land than over ocean for both the simulation with an interactive land scheme (middle column) and the simulation forced with surface fluxes (right‐hand column). Supplying skin temperature and surface fluxes reduces differences over ocean, while it increases differences over land.

The SCM simulations over land with the interactive land surface scheme contain identical parameterized processes to the full model. Therefore, the SCM biases in panel (b) indicate the bias in the SCM simulation due only to the approximations made in supplying the forcing. Over 9 days, these biases reach 1° to 2° over much of the troposphere. This is approximately 10% of the magnitude of the biases that are observed in section 6 when the SCM is forced with and compared to Cascade. For u and v, the biases reach approximately 10% those in the Cascade‐forced simulations (not shown), while for q the biases reach up to 20% of those in the Cascade‐forced simulations (not shown). This gives an indication of the uncertainty in our estimates of bias in the SCM due to the limitations of the proposed methodology.

The SCM simulations over land forced using surface‐sensible and latent heat fluxes have a larger bias in temperature than the simulations with the interactive land scheme, as shown in Figure 2(c). Biases in u and v are very similar between the two sets of simulations, whereas biases in q are also higher over land on supplying surface fluxes (not shown). This indicates an inconsistency in how the surface flux forcing is currently implemented in the SCM, which should be addressed in a future release of the SCM.

Over ocean points, the global IFS model includes a coupled wave model which is not present in the IFS SCM, such that over ocean, neither set of SCM simulations contain identical parameterized processes to the full model. Supplying surface‐sensible and latent fluxes reduces biases over ocean. The only noticeable bias is a cold bias at the tropopause, present in both sets of simulations, over both land and ocean points.

We, therefore, conclude that the proposed methodology is able to accurately estimate the tropospheric forcing profiles required by the SCM over ocean. Over land, errors are introduced into the forcing profiles. This is likely due to the varying topography, which will introduce errors into the estimates of the horizontal gradients of the prognostic variables. Nevertheless, the errors introduced in this way result in biases that are generally an order of magnitude smaller than those we wish to study. The basic philosophy behind our approach appears to be sound.

### 5.2 Impact of Vertical Interpolation Procedure

Having verified that it is possible to estimate the forcing profiles for an SCM from instantaneous model data, we consider whether it is possible to coarse grain a high‐resolution simulation to the resolution required for the SCM. We expect the biases measured in the SCM to be higher when compared to this coarse‐grained high‐resolution simulation than when compared to the global T639 IFS simulation. This is not because the input forcing data are poorer, but rather because the input data have a different set of biases compared with the IFS simulations, allowing us to measure the true biases in the SCM. These biases were masked when comparing the SCM to the global T639 IFS simulation, as the IFS SCM shares biases with its parent model.

We first consider the impact of the choice of interpolation procedure on the diagnosed biases of the IFS SCM by comparing the two methods outlined in section 4.1.1. While it is possible that the choice of interpolation will have no impact on the biases subsequently diagnosed in the IFS SCM, indicating a robustness in the coarse‐graining methodology, it is also possible that one of the two methods will produce a forcing data set that is more consistent with the SCM formulation. In this case, the biases will differ between the two methods: one of the two methods will allow for diagnoses of the true bias in the SCM with respect to the high‐resolution simulation, whereas the alternative method would introduce additional spurious biases into the SCM integration.

**Figure 3:**
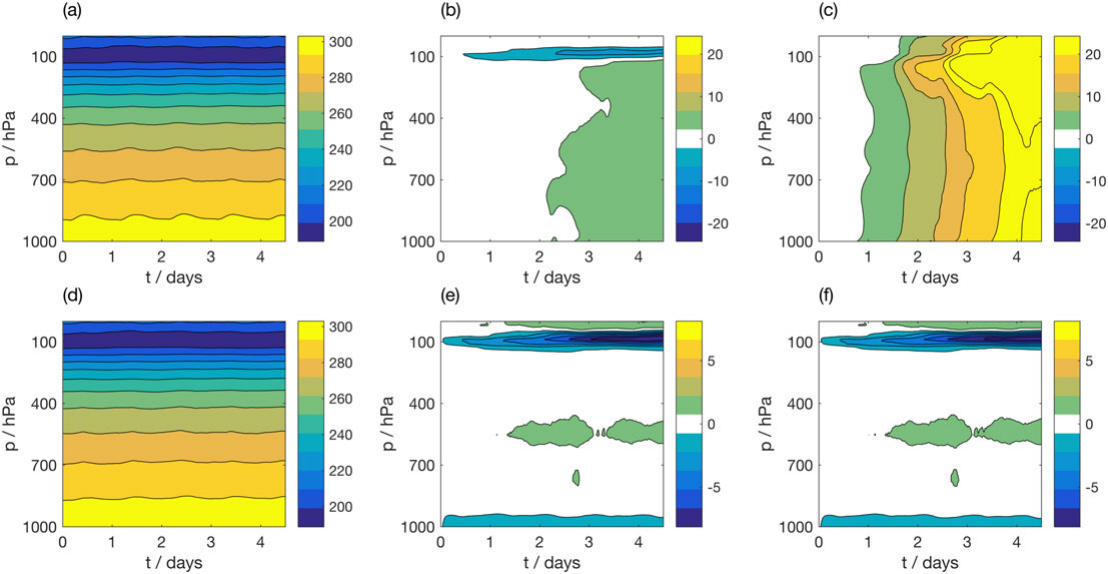
Evolution of temperature (K) as a function of height in coarse‐grained Cascade truth data, averaged over (a) land points and (d) ocean points in the Maritime Continent (MC) region. Bias in IFS SCM simulation over MC for the simulation with (b, e) forcing derived using Method 1 and (c, f) forcing derived using Method 2, over land and ocean points, respectively. IFS = Integrated Forecasting System; SCM = single‐column model.

Figure 3 shows the bias in forecast temperature as a function of lead time within the MC region, where the bias is considered separately for land and ocean points. The bias in each SCM integration is calculated by comparison with the coarse‐grained data set used to force the SCM integration. The mean evolution of temperature in Cascade is shown over (a) land and (d) ocean points. Note that Figures 3a and 3d show the coarse‐grained data following Method 1. The equivalent figures for Method 2 are largely indistinguishable so are not shown for brevity. Panel (b) shows the bias over land points for the SCM forced using Method 1, whereas panel (c) shows the bias for the SCM forced using Method 2. While both SCM integrations show an increasing warm bias with lead time over land, the SCM forced using Method 2 has a considerably larger bias (over 6 times larger at the surface). In contrast, the bias over ocean is extremely similar for the two integrations and remains small with increasing lead time. The biases in humidity, zonal, and meridional wind show similar characteristics between the two forcing methodologies, with a larger SCM bias observed for Method 2 over land points and similar biases over ocean points (not shown).

In section 5.1, a cold tropopause bias was identified as a *fingerprint* of the proposed methodology in the MC region, arising from the derivation of the forcing data sets from instantaneous prognostic fields. We expect to see this bias in SCM simulations in this region. While the cold tropopause bias is observed over both land and ocean points for Method 1, it is only observed over ocean for Method 2. Since this true bias cannot be diagnosed from the runs forced using Method 2, it appears that Method 1 produces forcing profiles that are more consistent with the SCM physics parameterizations. We therefore use Method 1 to derive the forcing profiles for the remainder of the paper. We note that these results may be sensitive to the relative resolutions of the grid, together with the assumptions about topography in the SCM of interest. In any case, it is clear that the profiles derived over ocean, or equivalently land regions with flat topography, are robust to the choices made during the coarse‐graining procedure.

### 5.3 Impact of Geostrophic Winds

**Figure 4:**
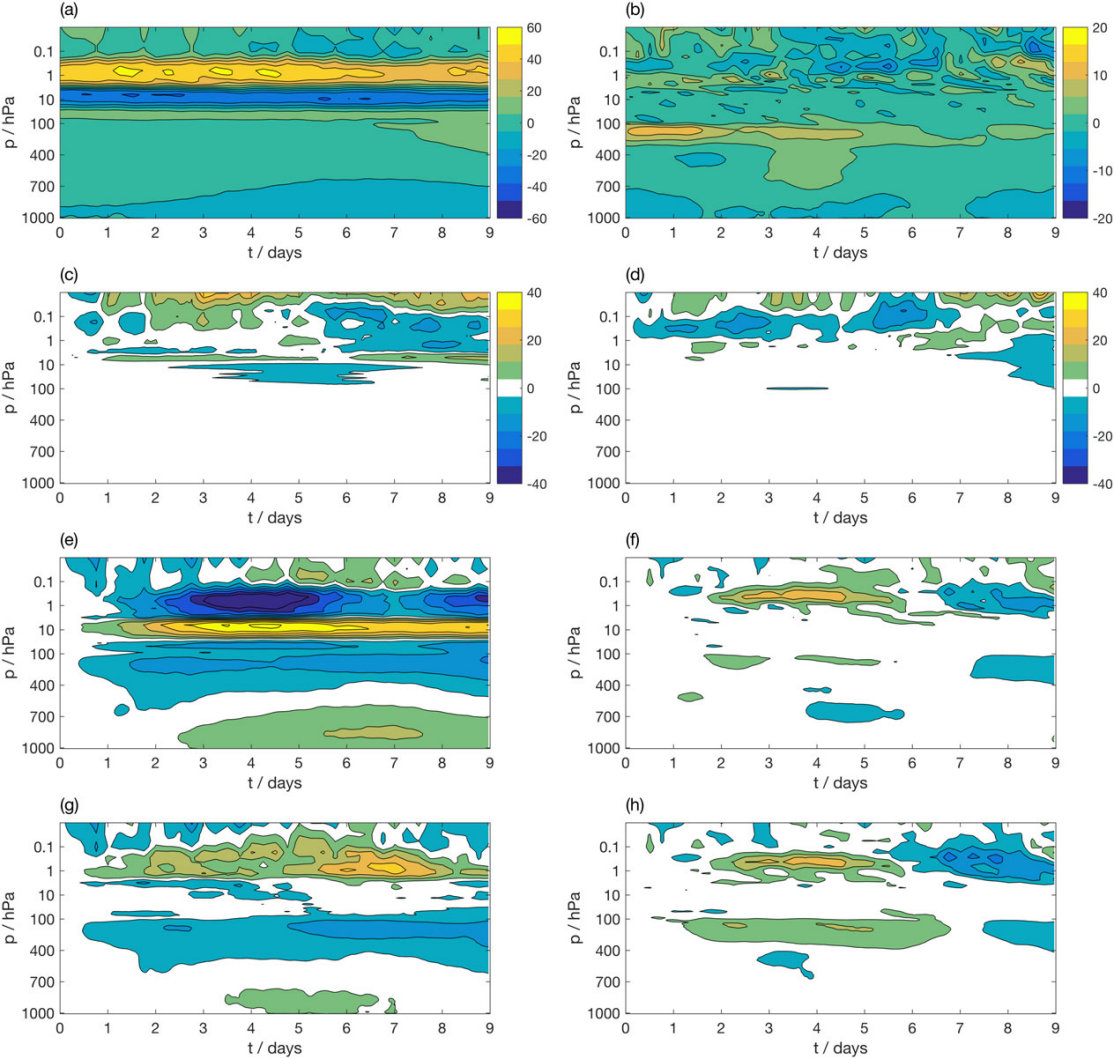
Evolution of zonal (left column) and meridional (right column) wind (m/s) as a function of height in (a, b) coarse‐grained Cascade truth data, averaged over West Pacific (WP) region. Bias in IFS SCM simulation over WP for the simulation with (c, d) derived geostrophic winds, (e, f) geostrophic winds set to zero, (g, h) geostrophic winds set to the large‐scale wind field. The color bar in (c) also corresponds to (e) and (g), and the color bar in (d) also corresponds to (f) and (h). IFS = Integrated Forecasting System; SCM = single‐column model.

The ability to consistently evaluate the geostrophic forcing is a key benefit of the proposed framework, in particular, due to the difficulty in estimating the geostrophic wind from field data (Svensson & Holtslag, 2009). This difficulty arises as geostrophic winds are not measured directly but must instead be calculated from pressure measurements taken over a sufficiently large area to allow estimation of a smooth pressure gradient (Baas et al., 2010). The difficulty in accessing reliable estimates of the geostrophic winds force SCM users to make approximations, such as assuming the geostrophic winds equal a constant value (e.g., Svensson et al., 2011; Zhu et al., 2005) or setting them equal to the large‐scale wind field (e.g., Brown et al., 2002; Dal Gesso et al., 2015). To assess the realism of the derived forcing, free‐running simulations forced with the derived geostrophic wind fields are compared to the extreme cases of setting the geostrophic winds to a constant (zero), or to the coarse‐grained large‐scale wind fields. The bias in the IFS SCM compared to the Cascade data set is evaluated over each region. Figure 4 shows the bias in zonal and meridional winds as a function of forecast lead time and model level over the WP region. The results for WP are representative of all regions studied, so results from the MC and WA are not shown for brevity.

The free‐running IFS SCM simulation forced with geostrophic winds from Cascade shows the lowest bias over the troposphere for both zonal and meridional wind. For meridional wind, the region was further split according to hemisphere to consider compensating biases; this exacerbated the difference between the simulations (not shown). Setting the geostrophic winds to zero had a more detrimental effect on IFS SCM forecast bias than setting the geostrophic winds to the large‐scale wind field. The constant value of zero was chosen as an extreme case. It is possible that selecting a different, spatially varying, constant value would be more appropriate and would produce better results. For all simulations, the biases in wind were larger in the upper atmosphere. Setting the geostrophic wind forcing to zero acted to slow down the stratospheric zonal wind jets, and using the incorrect forcing accelerated an anomalous cross‐equatorial flow. We note that the biases in other variables are largely unaffected by the chosen geostrophic wind forcing, including temperature and humidity profiles, and precipitation flux, which is reassuring for cloud process studies using the IFS SCM.

## 6 Benefits of the Proposed Methodology

Having assessed the validity of the proposed methodology, we turn our attention to consideration of potential benefits of using high‐resolution simulations to derive SCM forcing data sets, compared to using observational or analysis‐derived products.

### 6.1 Importance of Sampling

**Figure 5:**
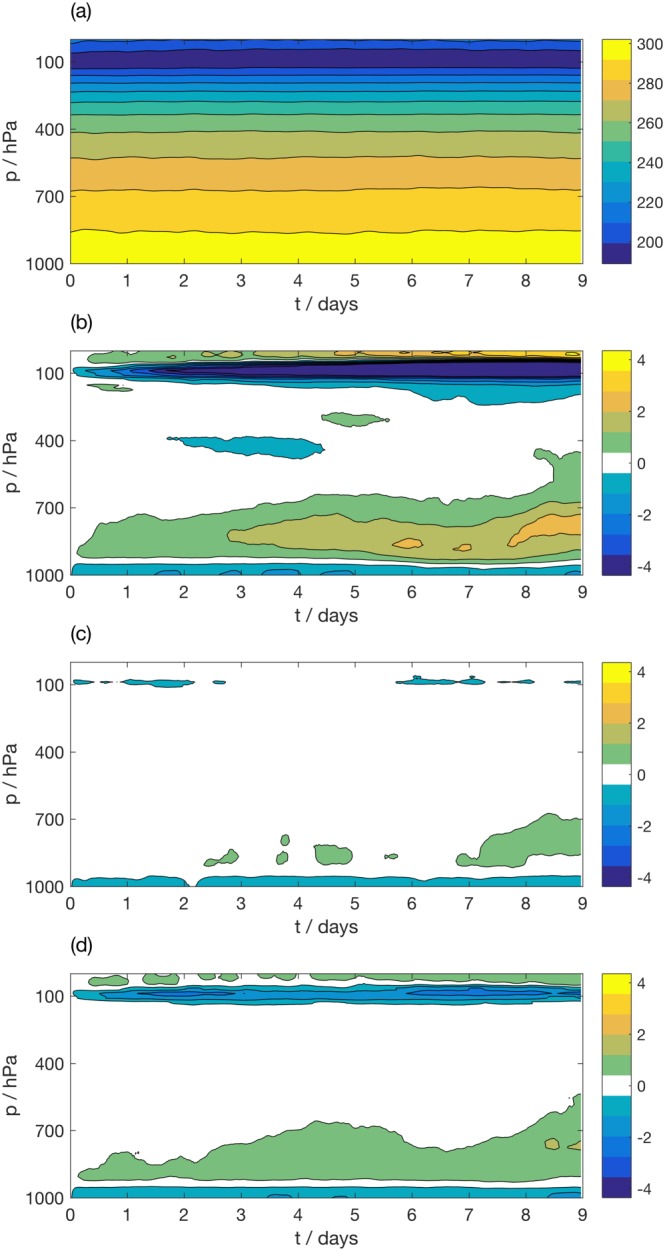
Evolution of temperature (K) as a function of height in (a) coarse‐grained Cascade truth data, averaged over West Pacific (WP) region. Bias in IFS SCM simulation over WP for the (b) free‐running simulation, (c) the strongly relaxed simulation τ=3 hr, and (d) the weakly relaxed simulation τ=24 hr. IFS = Integrated Forecasting System; SCM = single‐column model.

A key benefit of tiling many SCMs over a domain is the robust statistics that can be derived from analysis of the large number of simulations. If only one SCM integration were available, biases in the model may be less clearly identifiable. To demonstrate the usefulness of the proposed approach, we consider a series of experiments in which the model's prognostic variables (temperature, humidity, zonal and meridional wind) are relaxed toward the state derived from the Cascade simulation. This will minimize the drift away from a particular case of interest, and reduce the bias in the SCM integration. Two relaxation timescales, *τ*, are considered: 3 hr, and 24 hr. Figure 5 shows the impact of relaxation on temperature (*T*) for the WP region. As expected, when averaged over the region, the biases in temperature are smaller in the simulations that are relaxed toward Cascade. The free running simulation develops a warm bias of up to 3 K above the boundary layer. The relaxed runs show a greatly reduced bias.

**Figure 6:**
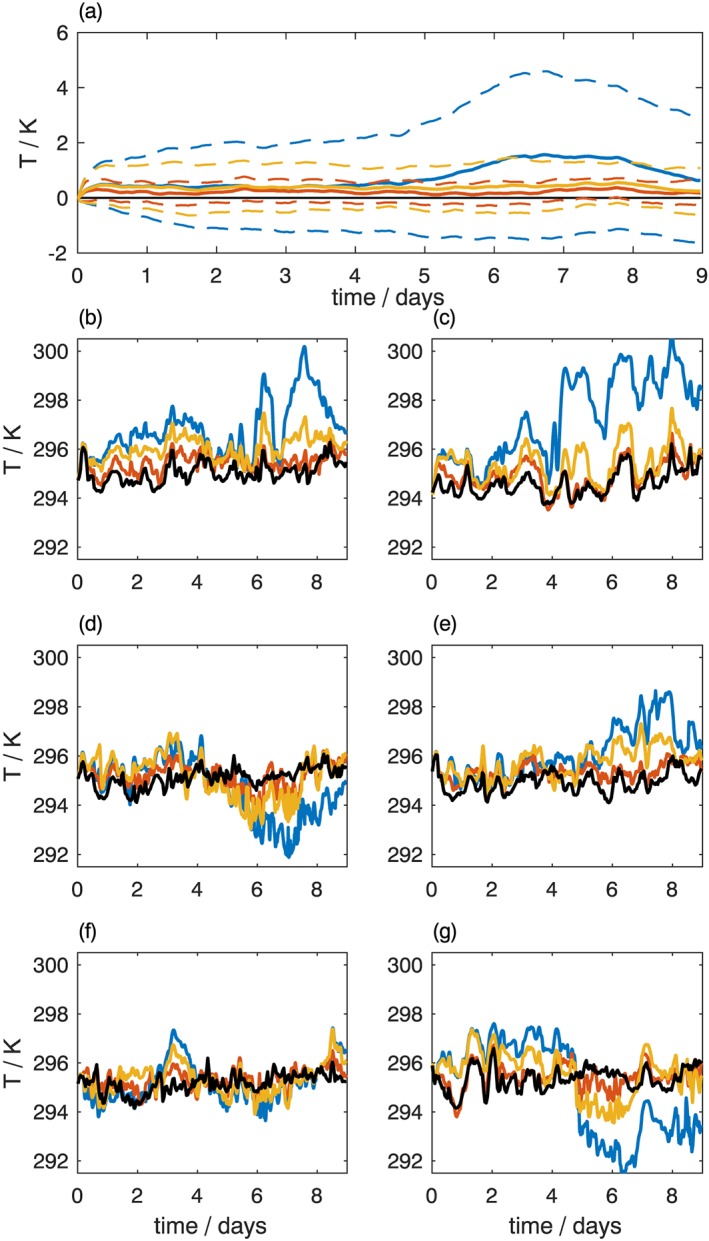
Evolution of temperature (K) at 925 hPa in the West Pacific region. (a) Mean (solid lines) and standard deviation (dashed lines) of difference between IFS SCM simulations and Cascade evaluated across region. (b)–(g) Time series from individual columns taken from within the region. For each figure, black = Cascade; blue = free‐running IFS SCM; red = strongly relaxed IFS SCM; yellow = weakly relaxed IFS SCM. IFS = Integrated Forecasting System; SCM = single‐column model.

Figure 6 demonstrates the variability in the evolution of temperature at 925 hPa across the three sets of SCM integrations within the WP region. As observed in Figure 5, on average the free‐running simulations show a warming trend over the period compared to the relaxed SCM simulations, as shown in panel (a) by the mean and standard deviation of the SCM biases evaluated across grid points within the domain. However, this is not ubiquitous. When considered independently, the individual grid points shown in panels (b)–(g) show large differences. Even over this limited domain, there is considerable spatial variability, with the largest warming biases observed south of 1^∘^N, and east of 164^∘^E (not shown). From a single grid point, it is difficult to draw conclusions about systematic biases in a forecast model.

### 6.2 Evaluation of State‐Dependent Model Biases

**Figure 7:**
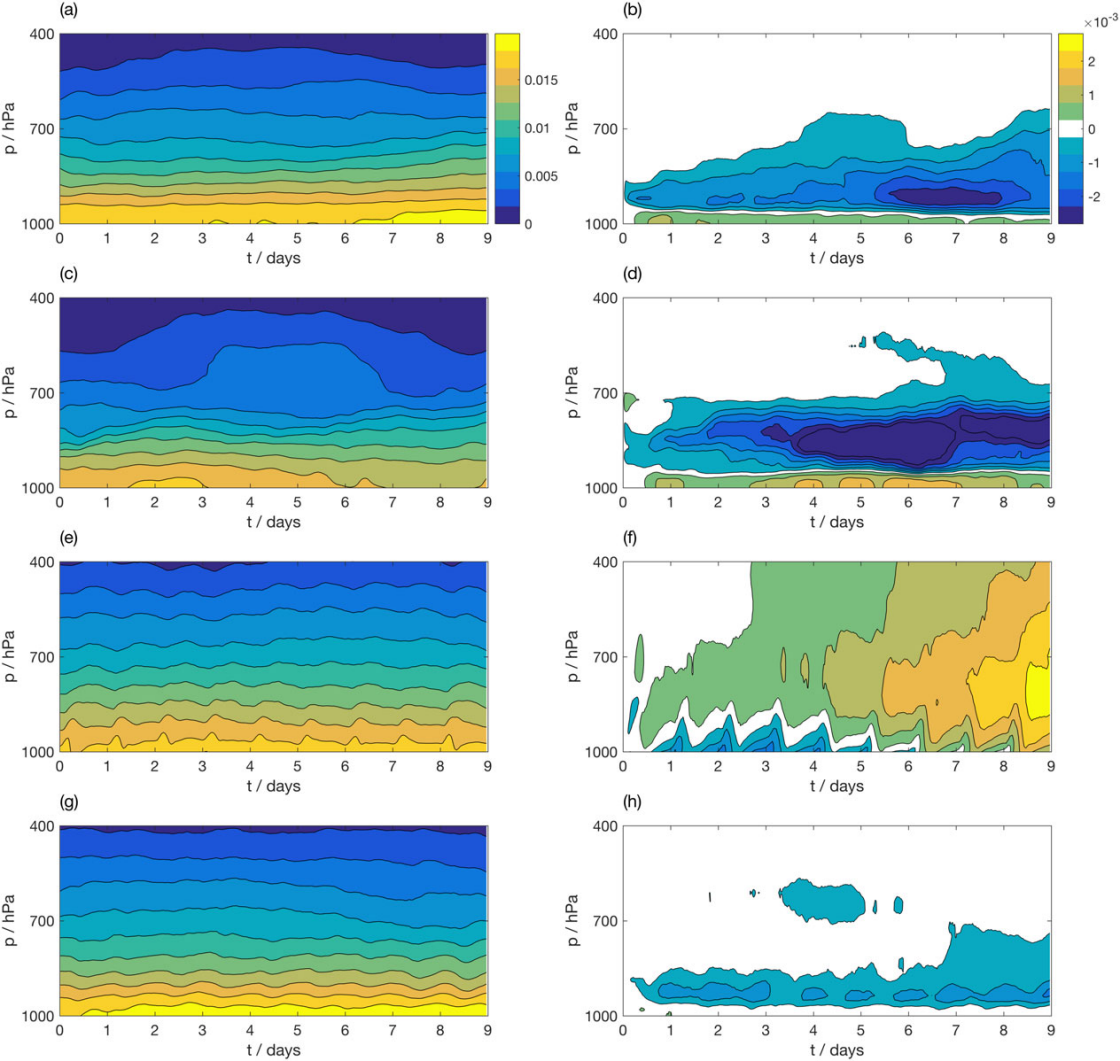
Evolution of water vapor mixing ratio (kg/kg) as a function of height averaged within (a, b) the West Pacific region; (c, d) the West Australia region; (e, f) land points within the MC region; (g–h) ocean points within the Maritime Continent region. (left column) Coarse‐grained Cascade truth data. (right column) Bias in the free‐running IFS SCM simulation. IFS = Integrated Forecasting System; SCM = single‐column model.

The large spatial domain covered by the Cascade data set allows for state‐dependent model biases to be evaluated. To demonstrate this, the evolution of humidity was evaluated in each region identified in Table 1. Figure 7 shows the evolution of water vapor mixing ratio in the coarse‐grained Cascade truth data (left column) and the bias in the SCM for each region. In the WP region (top row), the simulation shows a significant drying of the lower troposphere (950–700 hPa) accompanied by a moistening of the boundary layer. The IFS seems to prefer a drier troposphere than the MetUM Cascade simulation. It is important to verify that this is a true bias in the IFS and not a bias in the MetUM Cascade simulation: the Cascade simulation does not exhibit significant biases in tropospheric humidity compared to ERA Interim reanalysis data, indicating this is a true bias in the IFS (not shown). The WA region (second row) shows a similar pattern of bias to the WP region, even though the Cascade simulation (panel c) shows a substantially different evolution of humidity in that region. The bias in the MC region was decomposed into a bias over ocean and over land points. Over ocean (panels g and h), the bias is similar in the MC region to the WP region, showing the development of a dry bias above the boundary layer. However, the bias is less severe in this region compared to the WP region, and it also displays a strong diurnal cycle which was not evident in the WP. On the other hand, the biases over MC land regions (panels e and f) are markedly different. We observe a diurnally varying drying of the boundary layer. We also observe a strong moistening across the depth of the troposphere, which dominates the bias from 3 to 4 days into the simulation.

**Figure 8:**
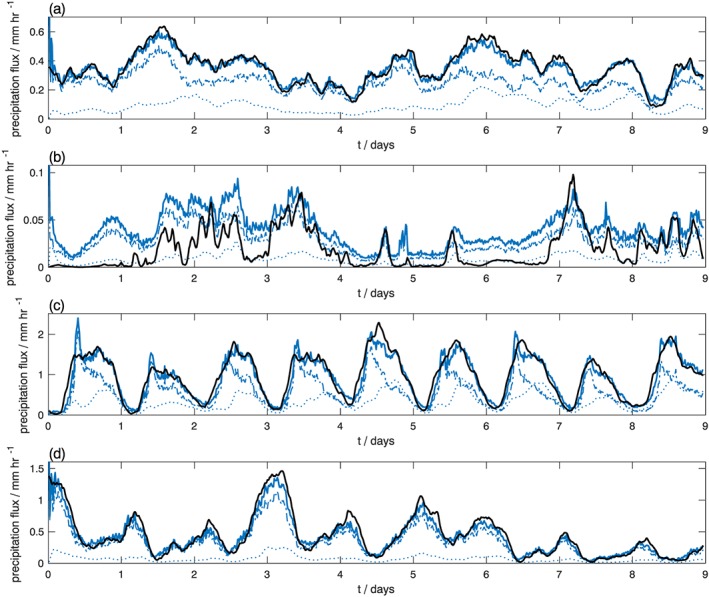
Precipitation flux as a function of lead time averaged over points within (a) the West Pacific region; (b) the West Australia region; (c) land points within the Maritime Continent region; (d) ocean points within the Maritime Continent region. Black line: precipitation from Cascade. Blue lines = precipitation from free‐running IFS SCM, where the solid, dashed, and dotted lines are total, convective, and stratiform precipitation, respectively. The Cascade precipitation has been scaled by 0.92 to remove the bias in the Cascade precipitation.

The domain‐averaged precipitation is shown for each region in Figure 8. Over ocean, a strong spin‐up of the IFS SCM is observed during the first hour of the simulation. During the spin‐up, the precipitation flux is higher than at later lead times and significantly greater than that indicated by Cascade. This high‐precipitation flux coincides with a slight rapid drying of the troposphere in the IFS SCM. By decomposing into convective and stratiform precipitation, it is evident that the initial enhanced precipitation is due to excess precipitation from the convection scheme. The large‐scale water processes scheme, which diagnoses stratiform precipitation, also shows a spin‐up over 4–6 time steps, but the spin‐up is in the opposite sense, with precipitation increasing from zero to a stable level. These contrasting spin‐up characteristics for convective and stratiform precipitation are observed over ocean points in all three regions considered. At later lead times, the IFS SCM precipitation flux in both WP and MC regions is a good match to that from Cascade, since the precipitation flux is likely largely determined by the prescribed large‐scale moisture convergence (e.g., Holloway et al., 2012).

The WA region was selected as a region with less convective activity than the WP and MC regions. In this region, precipitation biases vary greatly from model to model (Dai, 2006). Figure 8b shows that in this region, the SCM precipitation flux is lower than in the other regions. Stratiform precipitation accounts for approximately one quarter of the total precipitation. The match between the domain‐averaged precipitation flux for the Cascade data set and the SCM is poorer here than in other regions, with the SCM predicting higher precipitation than observed. This is because the SCM has a tendency to produce excessive light intensity precipitation compared to Cascade, while Holloway et al. (2012) demonstrated that the Cascade simulation has a distribution of precipitation rates similar to Tropical Rainfall Measuring Mission. Despite the reduced convective activity in this region, a strong spin‐up in precipitation flux is still observed.

Over land points in the MC region (panel c), the mean precipitation displays a strong diurnal cycle in both Cascade and the IFS SCM, with no initial spin‐up observed. The precipitation flux seems to be insensitive to the significant moistening of the troposphere toward the end of the simulation, again, because the precipitation flux is largely controlled by the applied forcing in an SCM framework. A diurnal cycle is also observed over ocean points in the MC region, out of phase with that over land, as identified in observations (Mori et al., 2004).

The SCM setup employed here removes the feedback between the parameterized physics and the model dynamics: advected tendencies are prescribed from Cascade. However, in a full GCM, the significant tropospheric moistening (drying) of the atmosphere over MC land (ocean) regions would impact the advected moisture tendencies in those regions, with an associated impact on precipitation. One would expect these biases to result in enhanced precipitation over land and suppressed precipitation over ocean regions. In fact, these biases have been documented in a range of forecast models (Love et al., 2011; Ulate et al., 2014). The source of this precipitation bias can therefore be investigated in this experimental setup.

### 6.3 Comparison with ECMWF Operational Analysis

By forcing an SCM using a high‐resolution simulation, we hope to identify shortcomings in the parameterized representation of physical processes such as clouds and convection in the SCM. Such high‐resolution simulations have been shown to capture these important physical processes. However, an existing alternative to our proposed approach is to use reanalysis products to derive the required forcing fields. Similar to our proposed methodology, the use of analysis products allows for excellent spatial and temporal sampling, such that an SCM may be tested in many weather regimes across many locations. However, the analysis blends a parameterized model simulation with observational data, so it is possible that physical processes such as clouds and convection are less well represented in an analysis data set than in a high‐resolution simulation. This will affect the diagnosis of biases in the SCM.

Therefore, as a final test, we compare forcing the IFS SCM using coarse‐grained Cascade data with forcing the IFS SCM using fields derived from the ECMWF operational analysis. Note that this is the same ECMWF operational analysis as used to drive the MetUM to produce the high‐resolution Cascade simulation. The resolution of the analysis is already as required for the SCM (T639), and all model levels are archived, but it is only available every 6 hr (at 00, 06, 12, 18 Coordinated Universal Time (UTC) time). The operational analysis is therefore linearly interpolated to a resolution of 15 min before the Cascade coarse‐graining scripts are used to derive the advective forcing fields. The resultant forcing data set was used to drive the IFS SCM in an analogous way to the Cascade data and the resultant SCM evolution compared to the analysis data set.

**Figure 9:**
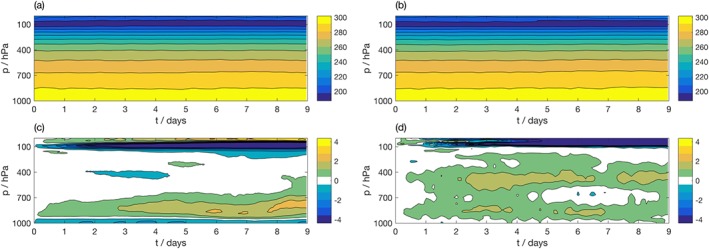
Temperature (K) as a function of height averaged over points within the West Pacific region. (a) Coarse‐grained Cascade truth data. (b) Operational analysis truth data. (c) Bias in the free‐running IFS SCM simulation forced using Cascade data, compared to Cascade data. (d) Bias in the free‐running IFS SCM simulation forced using ECMWF analysis data, compared to ECMWF analysis data. The color bars in (b) and (d) correspond to (a) and (c), respectively. Panels (a) and (c) are amplified from Figure 5. IFS = Integrated Forecasting System; SCM = single‐column model; ECMWF = European Centre for Medium‐Range Weather Forecast.

**Figure 10:**
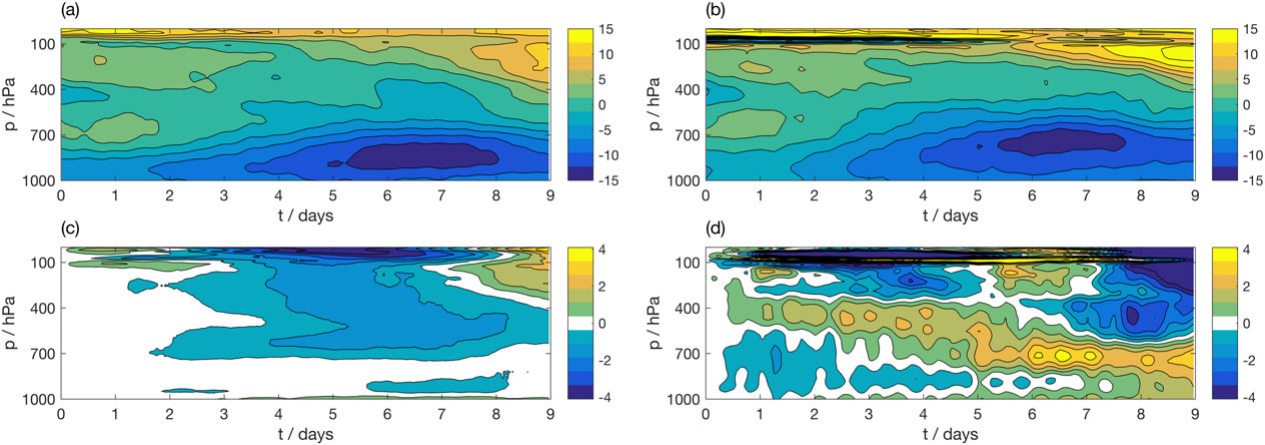
As for Figure 9, except for zonal wind (m/s) averaged over points within the West Pacific region. Panels (a) and (c) are amplified from Figure 4.

Figures 9 and 10 show the IFS SCM biases for T and u, respectively, over the WP region. Panels (a) and (b) show the mean profiles in the coarse‐grained forcing data from Cascade and ECMWF analysis, respectively, while panels (c) and (d) show the differences between the IFS SCM simulations and their respective target data sets, averaged over the region. While the evolution of the Cascade forcing profiles in (a) follows the analysis in (b) for both T and u, panels (c) and (d) show that the biases diagnosed in the IFS SCM are somewhat different for each forcing data set. In particular, the bias in the IFS SCM forced by the analysis shows high‐frequency beating with a period of 12 hr for both T and u. This behavior is spurious and due to the linear interpolation performed on the 6‐hourly operational analysis to derive the SCM forcing files. While the evolution of the SCM forced by analysis data is smooth, sharp features are present in the analysis‐derived input fields. These features are apparent when the bias shown in panel (d) is calculated. It would be possible to perform a more sophisticated interpolation procedure, though it is likely this will not remove the high‐frequency behavior completely. Furthermore, linear interpolation is the default interpolation scheme used by the IFS SCM if the specified time step is smaller than the time resolution of the supplied forcing files.

The biases in temperature, shown in Figure 9, give a similar overall impression for the Cascade and analysis‐driven integrations: a warm bias in the troposphere between 950 hPa and 400 hPa. Similar agreement is observed for biases in *q* for the WP region (not shown). This agreement is encouraging, as it indicates that the first‐order conclusions are not dependent on the target/forcing data set. However, the bias in the IFS SCM driven by ECMWF analysis does not grow in time, whereas the bias in the IFS SCM simulation driven by Cascade continues to grow and has not saturated by the end of the 9‐day integration. The warm mid‐tropospheric bias in the Cascade‐forced simulation is colocated with a dry bias, while the cold bias below 950 hPa is associated with a moist bias. A similar association can be identified in the analysis‐driven simulation, but the signal is obscured by the interpolation issues.

However, the wind fields are different between the Cascade and analysis driven integrations, despite the similarities in the forcing data shown in Figures 10a and 10b. The Cascade simulation was chosen to coincide with a period of convective activity, with a large MJO event crossing the domain during the simulation window. The high–resolution Cascade data set explicitly simulates organized convection, including the influence of mesoscale convective systems on the large‐scale dynamic fields. We, therefore, expect the wind fields in the Cascade data set to accurately reflect the state of the observed winds. It is not known how accurately the ECMWF analysis captured the details of this event, as conventional convection parameterization schemes are unable to represent organized convection. In order to further understand the biases in wind in the SCM, the biases in the forcing data sets need to be further analyzed. Nevertheless, it is clear that SCM wind fields are more sensitive to the data set chosen to drive the SCM than temperature and humidity, such that care must be taken when attempting to identify the effect of model error on zonal and meridional wind.

In general, it is expected that the IFS SCM biases would appear smaller when compared to ECMWF operational analysis than to the coarse‐grained Cascade data set. The operational analysis is a blend of observations with ECMWF model forecasts, and is therefore potentially biased compared to observations (Nagarajan & Aiyyer, 2004; Nuret & Chong, 1996). If the source of the bias is the model's physical parameterization schemes, the analysis bias will be in the same direction as the bias in the IFS SCM. This is observed to some extent: the warm, dry, midtropospheric bias increased in time in the Cascade‐driven simulation but not in the analysis‐driven simulation. However, the wind fields show no such bias developing, and, in fact, the analysis‐driven simulations have larger biases than the Cascade‐driven simulations for *u*.

## 7 Discussion and Concluding Remarks

In this paper we propose and describe the implementation of a new approach for deriving SCM forcing data sets. Existing high‐resolution data sets, which permit many physical processes of interest and resolve mesoscale circulations, are coarse‐grained to the resolution of a forecast model and thereby used to derive the required forcing data for an SCM. The utilization of existing data sets in this way increases their impact, making them accessible and usable by a wider community. The spatial domain of these data sets means that they span many coarse‐scale grid boxes, allowing many SCMs to be run in parallel, which we have demonstrated allows for excellent sampling of a regime or region of interest. This allows for identification of model developments that give statistically significant improvements to forecasts. The benefits of running many SCMs over a domain compared to evaluating a parameterization scheme in a GCM are perfect parallelization and complete control over the dynamical forcing, allowing for explicit evaluation of the physical parameterization schemes. The chief drawback is the lack of feedback between the parameterized physics and the model dynamics.

We propose a methodology for deriving SCM initial conditions and forcing fields from such data sets. We apply the methodology to data from a high‐resolution, convection‐permitting simulation produced using the UK Met Office's Unified Model as part of the Cascade project. We use this data to drive the ECMWF IFS SCM.

We first perform a consistency test whereby prognostic variables from a global T639 IFS simulation are used to derive the forcing fields for the IFS SCM. Over ocean, the SCM simulations track the global model closely, indicating a consistency between the estimated forcing fields and the true dynamical forcing in the global model. Over land the SCM simulations drift from the global IFS simulation, likely because varying topography leads to errors in the estimates of horizontal gradients required for calculating the forcing fields. However, we note that the drift is small compared to the difference measured between the SCM and Cascade forcing data set.

We demonstrate that the proposed methodology allows for a good representation of the time‐varying geostrophic wind forcing, which is notoriously hard to derive from observations. We demonstrate the possibility of detecting regionally dependent systematic biases. This is difficult to achieve if only SCM forcing data sets derived from observations are used, due to the sparsity of suitable observation sites. In particular, the IFS SCM develops a strong moist bias over land in the MC, while nearby ocean grid points show a slight dry bias.

The availability of the high‐resolution model data in addition to the coarse‐grained fields allows for consideration of subgrid variability of fields of interest. For example, while we have not yet explored this possibility, it would be possible to calculate the distribution of moist static energy, convectively available potential energy, convective inhibition, and more, on the fine‐scale grid, before comparing to the fields evaluated on the coarse scale. This could inform convection parameterization development, especially related to the stochasticity of convection (Craig & Cohen, 2006; J. Lin & Neelin, 2000; Shutts & Palmer, 2007). More generally, this framework could be very useful in the development of stochastic parameterization schemes that seek to represent unresolved subgrid‐scale variability (Buizza et al., 1999; Palmer, 2001; Palmer et al., 2009). By comparing the evolution of the high‐resolution, convection‐permitting simulation with a lower‐resolution single‐column model forecast, the *error* in the forecast model can be measured. The ability to separate the physics parameterization schemes from the dynamics is very useful, as most stochastic schemes assume that model error is due only to the parameterized physics. The usefulness of SCM forcing data that includes an estimate of subgrid variability has been demonstrated by Tang and Zhang (2015) and Tang et al. (2017), who consider subgrid variability in forcing fields derived from measurements at the Atmospheric Radiation Measurement Southern Great Plains site. In particular, they highlight the utility of such data sets for deriving and evaluating scale‐aware parameterization schemes, since the SCM forcing files can be specified across a range of resolutions (Tang et al., 2017). Our model‐derived SCM forcing data set could also be used for such studies.

Importantly, the high‐resolution simulation resolves mesoscale circulations that are not resolved by global climate models and are also missing from contemporary convective parameterization schemes. It is known that such circulations are a prominent physical feature of the Maritime Continent, so biases due to the presence of these mesoscale features can be evaluated. It would be possible to evaluate the degree to which these large‐scale circulations can organize convection, for example, through comparison with sensitivity experiments that set horizontal advection terms to zero. By comparing the parameterized tendencies with the Cascade simulation, the importance of feedback from convection onto the mesoscale circulations can be assessed. It is possible that the proposed SCM framework could be used to inform new developments in convection parameterization, which include the impact of such circulation features (Moncrieff et al., 2017).

We compared IFS SCM simulations driven with coarse‐grained Cascade data to those driven using the ECMWF operational analysis. While the input prognostic fields look similar, the diagnosed biases differ in their details. The analysis data are archived only every 6 hr so must be interpolated to drive the IFS SCM. This interpolation introduces unavoidable spurious features into the forcing fields, which appear as high‐frequency biases in the IFS SCM simulation. While overall conclusions are consistent between the two forcing data sets for temperature and humidity, the wind biases had different characteristics. As discussed above, the Cascade simulation is able to capture the organization of convection, whereas the parameterization schemes in the analysis are unable to represent this. It is therefore possible that the mesoscale circulations are better represented in the Cascade model, allowing for more robust calculation of biases in the SCM. Since the analysis is a blend of IFS model forecasts with observations, it is likely to share the same biases as the IFS SCM, which could hinder their identification (Nuret & Chong, 1996), whereas using a high‐resolution simulation from a different source allows for identification of these biases. There are several additional benefits of using the Cascade‐derived forcing data set over that derived from the ECMWF analysis. Precipitation rates from the high‐resolution simulation can be directly compared with the IFS SCM, allowing for consistency in analyzing the SCM water budget. In contrast, precipitation is not available as an ECMWF analysis product but only as a short range forecast produced using a model with a much lower resolution than Cascade. Furthermore, the high‐resolution simulation allows for *measurements* of quantities that are hard to measure, such as the vertical distribution of liquid water and ice: this will be a focus of future studies.

Our technique of initializing an SCM using data from a different model is a similar technique to that employed in the *Transpose‐Atmospheric Model Intercomparison Project (T‐AMIP)* framework (Williams et al., 2013). For T‐AMIP, climate modeling centers involved in the fifth phase of the Climate Model Intercomparison Project (CMIP5) were asked to also submit 5 day hindcasts using their model initialized from the ECMWF analyses. While it can be difficult to identify errors in parameterization schemes from climate simulations, studying initial tendencies and short‐range forecasts can provide significant insight (e.g., Cavallo et al., 2016; Hannay et al., 2009; Medeiros et al., 2012; Phillips et al., 2004; Rodwell & Palmer, 2007; Williamson et al., 2005). Since not all climate modeling centers have data assimilation capabilities, initializing from a common analysis provided a consistent framework for the study. In the case of SCM intercomparison studies, we demonstrate that using a forcing data set derived from a high‐resolution simulation is a viable alternative to using a common analysis, with benefits over using an analysis product. Note that one key difference between the T‐AMIP approach and the SCM approach presented here is that the SCM is not only initialized but also forced with data from a different model.

One problem encountered when initializing a model from a nonnative starting state is the possibility of an *initial shock* as the forecast model moves rapidly toward its own attractor (Klocke & Rodwell, 2014; Mulholland et al., 2015). We only see evidence suggesting this behavior in humidity forecasts in the SCM, with an initial rapid drying and associated anomalously high precipitation rate over the first hour, which decrease to a stable rate. However, it is known that the global IFS exhibits a spin‐up in precipitation, similarly observed as a decrease in precipitation flux over the first few hours of the forecast from an anomalously high initial rate, with associated systematic moisture increments (Beljaars, 2005). In this case it is therefore difficult to separate any potential initial shock from known spin‐up issues in the model.

The high‐resolution simulation used to provide the coarse‐grained initial conditions and forcing fields for the IFS SCM is only a proxy for the truth. When evaluating an SCM in this way, it is important to be aware of biases in the high‐resolution simulation which could confuse the results. SCM forcing data sets derived from high‐resolution simulations should be used in combination with existing approaches: the SCM should also be evaluated for specific case studies against observational data. Nevertheless, we have demonstrated the potential for making use of existing high‐resolution simulations in SCM studies, and have indicated some of the benefits of using this methodology over the use of observationally derived or analysis data sets. Future work will focus on applications of this new methodology, with a particular focus on informing parameterization development.

## Appendix A IFS Equations of Motion

### A.1

The IFS is expressed in terms of the hybrid vertical coordinate, *η*(*p*,*p*_*s*_), where *η*(0,*p*_*s*_) = 1 and *η*(*p*_*s*_,*p*_*s*_) = 0. The pressure can therefore be expressed as *p*_*k*_=*A*_*k*_+*B*_*k*_ *p*_*s*_, with index *k* indicating model level and where the discrete variables *A*_*k*_ and *B*_*k*_ are functions of *η*. In this coordinate system, the primitive equations can be written in terms of nonspherical horizontal coordinates as the momentum equations,

(A1) $$\frac{\mathrm{\partial u}}{\mathrm{\partial t}}+v_{\eta }\nabla_{\eta }u+\dot{\eta }\frac{\mathrm{\partial u}}{\partial \eta }=\mathrm{fv}-{\left.\frac{\partial \phi }{\mathrm{\partial x}}\right|}_{\eta }-{\left.\frac{1}{\rho }\frac{\mathrm{\partial p}}{\mathrm{\partial x}}\right|}_{\eta }+P_{u}$$

(A2) $$\frac{\mathrm{\partial v}}{\mathrm{\partial t}}+v_{\eta }\nabla_{\eta }v+\dot{\eta }\frac{\mathrm{\partial v}}{\partial \eta }=-\mathrm{fu}{\left.-\frac{\partial \phi }{\mathrm{\partial y}}\right|}_{\eta }-{\left.\frac{1}{\rho }\frac{\mathrm{\partial p}}{\mathrm{\partial y}}\right|}_{\eta }+P_{v}$$

the hydrostatic equation,

(A3) $$\frac{\partial \phi }{\partial \eta }=-\frac{1}{\rho }\frac{\mathrm{\partial p}}{\partial \eta }$$

the continuity equation,

(A4) $$\frac{\partial }{\mathrm{\partial t}}\left(\frac{\mathrm{\partial p}}{\partial \eta }\right)+\nabla_{\eta }\left(v_{\eta }\frac{\mathrm{\partial p}}{\partial \eta }\right)+\frac{\partial }{\partial \eta }\left(\dot{\eta }\frac{\mathrm{\partial p}}{\partial \eta }\right)=0$$

the tracer continuity equation,

(A5) $$\frac{\partial }{\mathrm{\partial t}}q+v_{\eta }\nabla_{\eta }q+\dot{\eta }\frac{\mathrm{\partial q}}{\partial \eta }=P_{q}$$

and the thermodynamic equation,

(A6) $$\frac{\mathrm{\partial T}}{\mathrm{\partial t}}+v_{\eta }\nabla_{\eta }T+\dot{\eta }\frac{\mathrm{\partial T}}{\partial \eta }-\frac{\left(R/c_{p,\text{dry}}\right)T_{v}\omega }{\left(1+(c_{p,\text{vap}}/c_{p,\text{dry}}-1)q\right)p}=P_{T}$$

Here the subscript *η* indicates that the derivative is taken along a surface of constant *η*. The symbols have the following meanings: **v**_*η*_=(*u*,*v*) is the vector wind along *η* surfaces, *p* is the pressure, *f* is the Coriolis parameter, *ϕ* is the geopotential, *P*_*i*_ denotes the tendency in variable *i* due to the parameterization schemes, *q* is a tracer, *T* is the temperature, $\omega =\frac{\mathrm{dp}}{\mathrm{dt}}$ is the vertical velocity, *R* is the gas constant, and *c*_*p*_ is the heat capacity at constant pressure.

The IFS SCM therefore solves the following set of equations at each time step.

(A7) $$\frac{\mathrm{\partial u}}{\mathrm{\partial t}}+\dot{\eta }\frac{\mathrm{\partial u}}{\partial \eta }=f(v-v_{g})+P_{u}+F_{u}$$

(A8) $$\frac{\mathrm{\partial v}}{\mathrm{\partial t}}+\dot{\eta }\frac{\mathrm{\partial v}}{\partial \eta }=-f(u-u_{g})+P_{v}+F_{v}$$

(A9) $$\frac{\mathrm{\partial T}}{\mathrm{\partial t}}+\dot{\eta }\frac{\mathrm{\partial T}}{\partial \eta }=\frac{\mathrm{R\omega}}{c_{p}p}T+P_{T}+F_{T}$$

(A10) $$\frac{\mathrm{\partial q}}{\mathrm{\partial t}}+\dot{\eta }\frac{\mathrm{\partial q}}{\partial \eta }=P_{q}+F_{q}$$

(A11) $$\frac{\mathrm{\partial a}}{\mathrm{\partial t}}=P_{a}$$

(A12) $$\frac{\partial }{\mathrm{\partial t}}(l+i)=P_{\left(l+i\right)}$$

The horizontal pressure term in the momentum equations have been written in terms of the geostrophic winds, and the horizontal advection terms have been replaced by a forcing term, *F*_*i*_. Note that advection of cloud fraction, *a*, specific liquid water, *l*, and specific ice water, *i*, are not included in the SCM. The geostrophic winds and advective forcing can be specified by the user. The vertical advection in equations (A7) requires the vertical velocity in *η* coordinates, $\dot{\eta }$. If available, this field can be supplied to the SCM. Otherwise, it is approximated using the following relationship:

(A13) $${\left.\dot{\eta }\frac{\mathrm{\partial p}}{\partial \eta }\right|}_{p_{s}}\approx \omega$$

Table A1 contains an exhaustive list of boundary conditions and prognostic variables required by the IFS SCM. All variables must be specified between the SCM forecast start and end times. If the time discretization of the variables is different from the forecast time step, linear interpolation is utilized by the model to provide the intermediate forcing values. The prognostic variables are only used after initialization if the SCM is relaxed toward the supplied data.

**Table A1:** Input Fields Required by the Integrated Forecasting System Single‐Column Model

Variable	Units	IFS file name	Comment
Coordinates			
Time	s	time	Seconds since start of run
Reference date		date	Reference day and hour for start of run
Reference second	s	second	Seconds start of run is offset from reference date
Latitude	degrees	lat	
Longitude	degrees	lon	
Model levels		nlev	1 ≤ nlev ≤ 91
Model half levels		nlevp1	1 ≤ nlevp1 ≤ 92
Soil/sea ice levels		nlevs	1 ≤ nlevs ≤ 4
Pressure on full levels	Pa	pressure_f	
Pressure on half levels	Pa	pressure_h	
Height on full levels	m	height_f	
Height on half levels	m	height_h	
Prognostic variables			
Zonal wind	m/s		Vertical profile
Meridional wind	m/s	v	Vertical profile
Temperature	K	t	Vertical profile
Water vapor mixing ratio	kg/kg	q	Vertical profile
Liquid water mixing ratio	kg/kg	ql	Vertical profile
Ice water mixing ratio	kg/kg	qi	Vertical profile
Surface pressure	Pa	ps	
Cloud fraction	1	cloud_fraction	
Optional Prognostic Variables/Forcing
Skin temperature	K	t_skin	Specified if land surface scheme off
Skin reservoir water content	m	q_skin	Specified if land surface scheme off
Soil temperature	K	t_soil	Four levels, specified if land surface scheme off
Soil moisture	m	q_soil	Four levels, specified if land surface scheme off
Snow depth	m	snow	Specified if land surface scheme off
Forcing			
Omega	Pa/s	omega	Vertical profile
$\dot{\eta }\frac{dp}{d\eta }$	Pa/s	etadotdpdeta	Optional field, vertical profile
Geostrophic U wind	m/s	ug	Vertical profile
Geostrophic V wind	m/s	vg	Vertical profile
Advective T tendency	K/s	tadv	Vertical profile
Advective Q tendency	kg/kg/s	qadv	Vertical profile
Advective U tendency	m/s^2^	uadv	Vertical profile
Advective V tendency	m/s^2^	vadv	Vertical profile
Surface sensible heat flux	W/m^2^	sfc_sens_flx	Downward, specified if land surface scheme off
Surface latent heat flux	W/m^2^	sfc_lat_flx	Downward, specified if land surface scheme off
Boundary Conditions			
Background albedo	0–1	abedo	
Orography	m	orog	
Angle of subgrid orography	rad	anor	
Anisotrophy of subgrid orography		isor	
*σ* of subgrid orography		sdfor	
*σ* of slope of subgrid orog.		slor	
Heat roughness length	m	heat_rough	
Momentum roughness length	m	mom_rough	
High vegetation cover	0–1	high_veg_cover	
High vegetation type		high_veg_type	Index depicting type of vegetation
Low vegetation cover	0–1	low_veg_cover	
Low vegetation type		low_veg_type	Index depicting type of vegetation
Land‐sea mask	0 or 1	lsm	
Open sea surface temperature	K	open_sst	
Sea ice fraction		sea_ice_frct	
Temperature of sea ice	K	t_sea_ice	Four levels
Geopotential height at the surface	m^2^/s^2^	z_sfc	

## Appendix B Availability of Software and Data

### B.1

The software produced for this project are freely available to interested users, consisting of the following:

1. NCL software to produce OpenIFS forcing fields from a high‐resolution MetUM simulation and necessary ECMWF boundary files. https://github.com/aopp-pred/cg-cascade
2. “scmtiles”: Python software to deploy many independent SCMs over a domain. https://github.com/aopp-pred/scmtiles
3. “openifs‐scmtiles”: Python software to deploy the OpenIFS SCM using scmtiles. https://github.com/aopp-pred/openifs-scmtiles

On successful application to ECMWF for an OpenIFS license, we can also supply the code changes required to disable the interactive land scheme in the IFS SCM.

The processed coarse‐grained Cascade data sets required to drive the IFS SCM (Christensen et al., 2018) are archived at the NERC Centre for Environmental Data Analysis (http://catalogue.ceda.ac.uk/uuid/bf4fb57ac7f9461db27dab77c8c97cf2).
